# In Vivo Monitoring of Acute and Intermittent Fatigue in Sport Climbing Using Near-Infrared Spectroscopy Wearable Biosensors

**DOI:** 10.3390/sports11020037

**Published:** 2023-02-06

**Authors:** Carlo Dindorf, Eva Bartaguiz, Jonas Dully, Max Sprenger, Stephan Becker, Michael Fröhlich, Oliver Ludwig

**Affiliations:** Department of Sports Science, Rheinland-Pfälzische Technische Universität Kaiserslautern-Landau, 67663 Kaiserslautern, Germany

**Keywords:** climbing, exhaustion, muscle oxidative metabolism, fatigue, training, sports, human-machine interaction, wearables, fatigue

## Abstract

The objectification of acute fatigue (during isometric muscle contraction) and cumulative fatigue (due to multiple intermittent isometric muscle contractions) plays an important role in sport climbing. The data of 42 participants were used in the study. Climbing performance was operationalized using maximal climbing-specific holding time (CSHT) by performing dead hangs. The test started with an initial measurement of handgrip strength (HGS) followed by three intermittent measurements of CSHT and HGS. During the test, finger flexor muscle oxygen saturation (SmO_2_) was measured using a near-infrared spectroscopy wearable biosensor. Significant reductions in CSHT and HGS could be found (*p* < 0.001), which indicates that the consecutive maximal isometric holding introduces cumulative fatigue. The reduction in CSHT did not correlate with a reduction in HGS over multiple consecutive maximal dead hangs (*p* > 0.35). Furthermore, there were no significant differences in initial SmO_2_ level, SmO_2_ level at termination, SmO_2_ recovery, and mean negative slope of the SmO_2_ saturation reduction between the different measurements (*p* > 0.24). Significant differences were found between pre-, termination-, and recovery- (10 s after termination) SmO_2_ levels (*p* < 0.001). Therefore, monitoring acute fatigue using athletes’ termination SmO_2_ saturation seems promising. By contrast, the measurement of HGS and muscle oxygen metabolism seems inappropriate for monitoring cumulative fatigue during intermittent isometric climbing-specific muscle contractions.

## 1. Introduction

Sport climbing has experienced increasing professionalization and public interest in the recent past, culminating in its inclusion in the Olympic Games. The Olympic climbing competition consisted of the three sub-disciplines speed and lead climbing as well as bouldering. To compete for overall victory, athletes can no longer specialize in one discipline, instead, climbers must be more versatile, more resistant to fatigue, and have a good ability to recover. To enable coaches and athletes to deal with these complex requirements in the best possible way, the intensive scientific debate on the subject of sport climbing appears to be substantial. In this context, the present work focuses on the aspect of fatigue and its possible monitoring in sport climbing.

Climbing time to exhaustion is a major factor in climbing performance [1]. Climbing is characterized by isometric loading of the finger flexors. Therefore, the objectification of acute fatigue (caused during isometric muscle contraction) and cumulative fatigue (as a result of multiple intermittent isometric muscle contractions) of the finger flexor muscles might play an important role in training planning, monitoring, and competition, which highlights the research necessity. Measuring handgrip strength (HGS) by means of a hand dynamometer and the maximal climbing-specific holding time (CSHT) is often associated with climbing-specific performance [2,3,4]. As these parameters are easy to measure objectively, they might be suitable for monitoring fatigue. However, little research has been performed regarding the effects of fatigue on the HGS [5] as well as the CSHT.

The continuous monitoring of physiological signals and motion data using wearable devices or wearable biosensors is playing an important role in more and more fields of sports. Most wearables in climbing are designed to track motions, plan routes, and give feedback on the power, control, stability, and speed of a climber [6]. Furthermore, wearables are best placed on the forearm or the wrist so that they do not disturb the climber. However, in climbing, only a few applications of wearables exist (e.g., oxygen uptake and heart rate [7]; accelerometer [8]; inertial measurement units [9]), and it seems that there is still great potential in this area which has not been utilized so far.

One promising application, which seems feasible in climbing, is near-infrared spectroscopy (NIRS). NIRS is a non-invasive technique designed to monitor muscle oxygen saturation (SmO_2_) in vivo [10] and shows good results regarding validity and reliability [11]. This seems practical, as it provides insight into the fatigue of the muscles [12], is easy to attach, and is almost unnoticeable by the climber once they get used to it. It might be used as an indicator of training status in sport climbing and relates to the overall climbing performance [13], as well as possibly forming the basis for an objective feedback system of fatigue (e.g., of biceps brachii [12]). With the ability to provide direct feedback on the muscle oxygen metabolism while climbing, these wearables might be used to provide objective feedback on acute and cumulative fatigue, predict failure, or monitor and regulate the training in general. In the context of HGS and CSHT, however, to the best knowledge of the authors, NIRS has only been used in a few applications. Force production correlates negatively with muscle oxygenation (SmO₂) in the forearms of climbers when performing HIT training [14]. This shows that SmO₂ might be used to define fatigue and therefore help to control the training. Most studies using NIRS in climbing focus on experienced, regularly climbing participants (e.g., [14,15]). Little research has been conducted on less experienced or inexperienced participants.

In summary, it can be said that, according to the current state of research, the analysis of fatigue using SmO_2_ has been considered too little and too undifferentiated in the context of sport climbing. As the measurement of SmO_2_ gives an insight into the muscle metabolism of the muscles relevant for holding, it may be more informative than the sole measurement of maximum force or holding time as it has been mostly practiced so far. Information on muscle metabolism, for example, may be particularly relevant for the development of training programs. With this study, we also expect to gain knowledge about the regeneration behavior of the hand muscles during intermittent loads, as required in sport climbing. Because there is hardly any scientific information on people with little or no experience in climbing, this is a worthwhile target group. Consequently, this study addresses the above-mentioned research deficiencies, analyzing the following questions using participants with different climbing experience (from experienced, but non-professional participants, to non-climbing participants): (a) Do the maximal climbing-specific holding time (CSHT) and handgrip strength (HGS) reduce as a result of cumulative fatigue during multiple consecutive maximal dead hangs? Does the reduction in CSHT relate to the reduction in HGS over multiple consecutive maximal dead hangs? (b) Is there an interrelationship between acute fatigue and cumulative fatigue and muscle oxidative metabolism measured by the SmO_2_ level? (c) Do different levels of maximal CSHT relate to termination SmO_2_, basal SmO_2_ level, and the variability of the termination SmO_2_ level over multiple consecutive maximal dead hangs?

## 2. Materials and Methods

### 2.1. Participants and Data Acquisition

For answering our research questions, the sample size was a priori determined according to G*Power (version 3.1.9.2, Heinrich-Heine-Universität Düsseldorf, Germany) [16] with a power (1 − β) set to 0.90 (α = 0.05; f = 0.25), resulting in a minimum of 36 participants. Initially, 48 participants were interested in participating in the study. As a first step, participants who could not hold themselves in a stable position during the dead hang were excluded because of higher injury risk (see, e.g., [17]). Further participants who were ill or had acute and recent injuries in the upper extremities were not allowed to participate in this study. Consequently, 44 participants participated in the measurement. Two participants with missing data points or displacement of the sensor during the measurement were excluded. For final data analysis, data of 42 healthy participants were used (sex: 22 female, 20 male; age: 22.45 ± 3.13 years; height: 173.17 ± 9.05 cm; weight: 70.55 ± 12.35 kg; body fat: 20.52 ± 8.36%; 10 participants were previous experienced in sport climbing or bouldering). The study was approved by the ethical committee of the university (reference number 55) and met the criteria of the Declaration of Helsinki [18]. The informed consent and permission to publish any results of the study of each participant was signed. The participants were instructed to participate in a rested state and therefore not perform any intense activities in the course of two days before the study.

### 2.2. General Measurement Procedure

**Figure 1:**
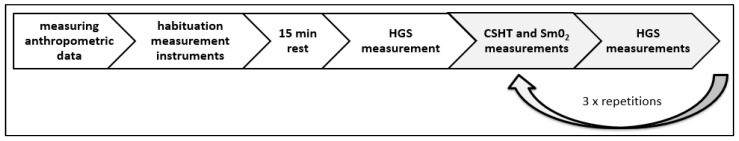
Schematic workflow chart. CSHT = maximal climbing-specific holding time; HGS = handgrip strength; SmO_2_ = finger flexor muscle oxygen saturation.

A schematic workflow of this study is presented in Figure 1. Before the study, each participant underwent a bioelectric impedance measurement to quantify the body composition using a bioelectric scale (InBody 770, InBody Europe, Eschborn, Germany). Afterwards, a phase of getting used to the fingerboard took place. For this purpose, the participants were instructed to hang on the fingerboard two times, with the termination of each hanging before subjective perception of fatigue. Afterwards, before the actual measurements started, a 15-minute-long break was ensured.

**Figure 2:**
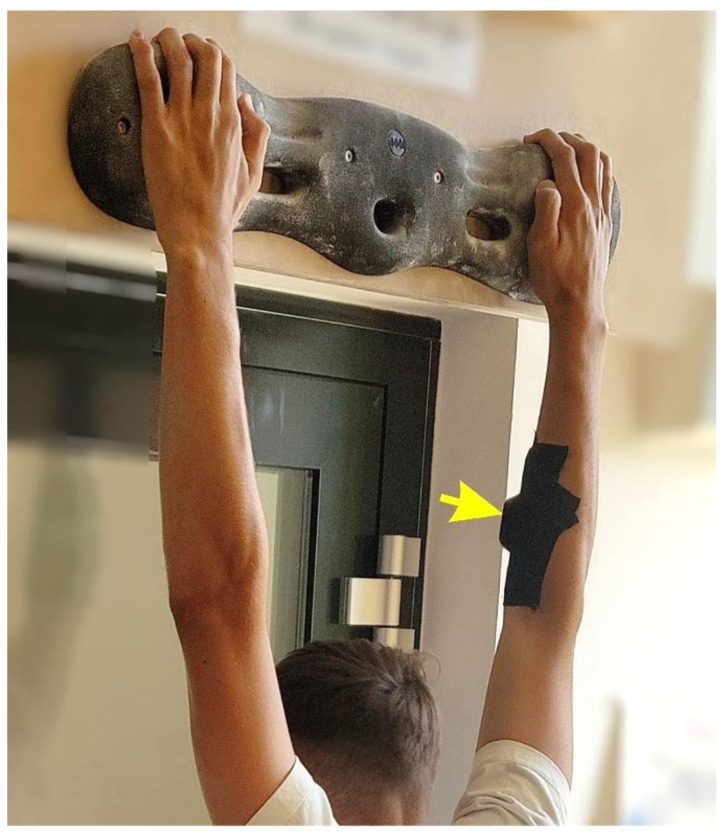
Hand positioning on the fingerboard and placement of the O₂-Sensor (arrow).

The maximal climbing-specific holding time (CSHT) was measured three times consecutively (these measurement phases are called TOM1, TOM2, and TOM3 in the following) with about 90 s in between to assess the influence of fatigue. The maximal CSHT was determined by measuring the hanging time on a 4-centimeter-deep crimp with rounded edges and structuring as is typically found on a hold in a climbing gym of a MOON fingerboard (Moon Climbing Limited, Sheffield, England) without touching the ground. Therefore, participants held a dead hang position with straight arms with the feet lifted at least 20 cm from the ground (depending on body height) on clean holds with loose magnesium on the fingers as commonly practiced in climbing to increase friction [19]. The participants were not allowed to use their thumb while hanging but could use three to four fingers depending on individual preference (see Figure 2).

The time between the CSHT trials was used to measure handgrip strength (HGS) using a Jamar hydraulic hand dynamometer (JLW Instruments, Chicago, IL, USA). Additionally, the HGS was measured before the begin of the treatment (this time of measurement is called TOM0 in the following). Each hand was tested two times for each test run, starting with the side of handedness. Following [20], the maximum value achieved with either hand was used as a person’s isometric maximal HGS. The measurements were carried out based on the instructions for the determination of the reference values according to [21].

During the whole process, the muscle oxygen level was continuously recorded using the Moxy sensor, which uses light from about 670 to 810 nm for measuring the SmO₂ level (Moxy Monitor, Huttchinson, MN, USA). In accordance with [22], the sensor was fixed at the forearm of the handedness using Kinesio tape. The Moxy sensor was placed over the muscle bellies of *m. flexor carpi radialis* and *m. palmaris longus* by drawing an imaginary straight line from the middle of the wrist to the medial epicondyle of the humerus. The recording interval was set to two seconds. Data were smoothed using a ten second moving average with removal of the delay with window length *N* according to (*N* − 1)^−2^.

For further statistical analysis, the following parameters were extracted based on the waveform data of every participant and fatigue treatment: initial SmO₂ directly before hanging, SmO_2_ at the termination time point, and SmO_2_ ten seconds after termination time (recovery). Additionally, the average slope of the SmO_2_ reduction was calculated by dividing the difference of initial and the termination SmO_2_ level by the CSHT for each time of measurement. Furthermore, the variability in termination SmO_2_ level over the different times of measurement was analyzed using the standard deviation.

### 2.3. Statistical Analysis

A repeated measures ANOVA was used to check changes over the times of measurements and differences for the SmO_2_ parameters (research question b). The effect size is presented by the partial eta squared (η_p_^2^). Relationships between the reduction in CSHT and the reduction in HGS (research question a) as well as relationships between different levels of the maximal CSHT with the mentioned SmO_2_ parameters (research question c) were evaluated using the Pearson correlation. For all statistical tests, necessary requirements were checked and could be assumed. Greenhouse–Geisser adjustments were made to correct violations of sphericity. Bonferroni correction was performed for post hoc testing. Statistical analysis was performed using IBM SPSS (version 25, SPSS Inc. Chicago, IL, USA).

## 3. Results

### 3.1. Research Question (a): Effects of Cumulative Fatigue on Maximal CSHT and HGS

**Figure 3:**
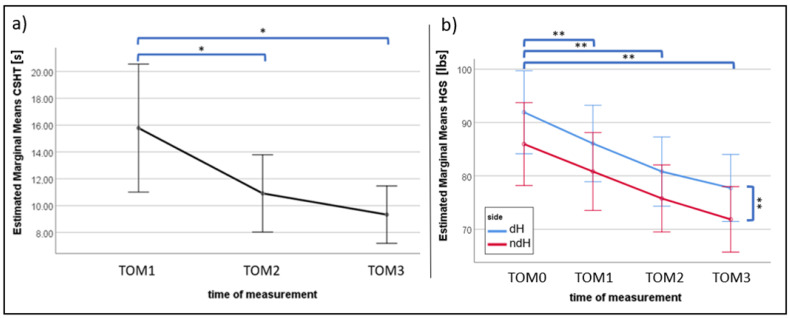
Estimated marginal means (means extracted from the statistical model) for (**a**) maximal climbing-specific holding time (CSHT) and (**b**) handgrip strength (HGS) during the different measurements (TOM0 = measurement before treatment, TOM1–TOM3 = measurements over the three repeated dead hangs on the fingerboard). Error bars indicate the confidence interval. dH = dominant hand, ndH non-dominant hand; * *p* < 0.05; ** = *p* < 0.001.

Both CSHT (F(1.29,60.72) = 12.43, *p* < 0.001, η_p_^2^ = 0.21) and HGS (F(2,28) = 35.40, *p* < 0.001, η_p_^2^ = 0.72) decreased significantly over the measurement time points, with higher values for the dominant hand compared to the non-dominant hand for HGS. The results of the post hoc tests are presented in Figure 3.

The reduction in CSHT and the reduction in HGS between measuring time points 1 and 2 (r = −0.01, *p* = 0.96, n = 42) as well as 2 and 3 (r = −0.14, *p* = 0.35, n = 42) do not significantly correlate.

### 3.2. Research Question (b): Effects of Acute and Cumulative Fatigue on Muscle Oxidative Metabolism

**Figure 4:**
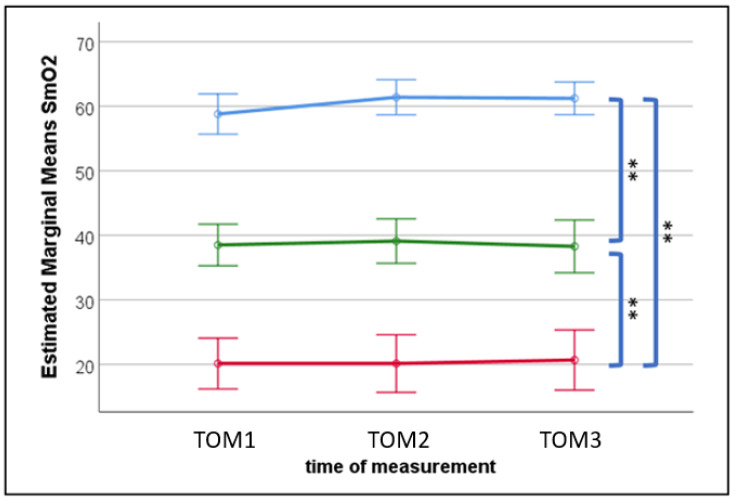
Differences in SmO_2_ level between pre- (blue), termination- (red), and recovery- (green) SmO_2_ saturation level and the three different measurement time points. Error bars indicate 95% confidence interval. ** = *p* < 0.001.

Repeated ANOVA results showed that there are significant differences between pre-SmO_2_ level, termination-SmO_2_ level, and recovery (10 sec after termination)-SmO_2_ level (F(1.47,60.05) = 284.33, *p* < 0.001, η_p_^2^ = 0.87; see also Figure 4). However, pre-, termination-, and recovery-SmO_2_ levels do not differ between the different measuring times TOM1, TOM2, and TOM3 (F(2, 82) = 0.54, *p* = 0.58). Furthermore, no interaction could be found (F(2.45,100.60) = 0.79, *p* = 0.48). The mean negative slope of the SmO_2_ reduction does not change significantly over the three measuring time points (F(2,82) = 1.46, *p* = 0.24).

### 3.3. Research Question (c): Relationships between Different Levels of Maximal CSHTs and SmO_2_ Parameters

**Figure 5:**
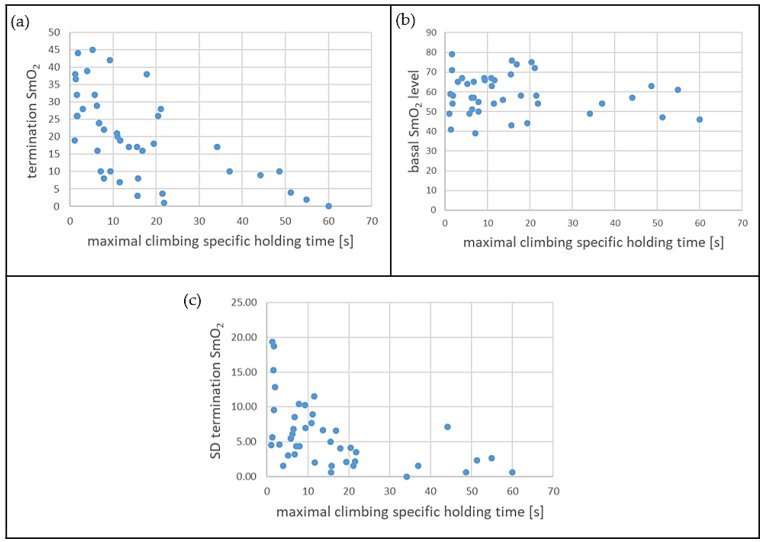
(**a**) SmO_2_ value at the first termination event, as well as (**b**) standard deviation (SD) of the minimal SmO_2_ level of the three measurements, and (**c**) basal SmO_2_ before the first measurement.

**Figure 6:**
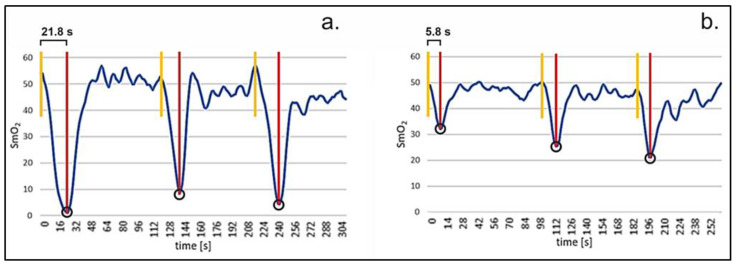
SmO_2_ profiles of an exemplary participant with relatively long CSHT (**a**) and a participant with short CSHT (**b**) over the whole measuring procedure. The yellow vertical lines represent the starting points (initial SmO_2_ level) of the three repeated dead hangs at TOM1, TOM2, and TOM3 on the fingerboard, and the red lines indicate fall off. The participant with higher CSHT is draining the SmO_2_ level more deeply for all three repeated dead hangs (SmO_2_ when falling down marked with O). TOM = time of measurement.

The relationships between different levels of maximal CSHTs and SmO_2_ parameters are presented in Figure 5. The CSHT and the basal SmO_2_ level do not correlate significantly (r = −0.15, *p* = 0.34, n = 42), which means that participants with longer CSHTs do not appear to have higher basal SmO_2_ levels. However, there is a correlation between the CSHT and the minimum SmO_2_ value achieved at the time of termination (r = −0.61, *p* < 0.001, n = 42). Furthermore, the CSHT correlates with the variability in SmO_2_ level at the time of termination (r = −0.51, *p* < 0.001, n = 42). Corresponding exemplary waveform data of one participant with a relatively long CSHT and one participant with a relatively short CSHT are presented in Figure 6.

## 4. Discussion

The significant difference between the initial (TOM0) and first (TOM1) measurement of the HGS as well as between the first (TOM1) and second (TOM2) measurement of the CSHT shows that consecutive maximal isometric holding induces cumulative fatigue in the finger flexors. However, between the measurements afterwards, no significant differences were found for both HGS and CSHT. This could be related to the regeneration time provided, which could just be sufficient to counteract a further decrease in CSHT or HGS.

Even though the CSHT decreases significantly during cumulative fatigue, the analyzed SmO_2_ levels (pre, termination, recovery) do not change significantly over the different times of measurement. Furthermore, no visible changes could be detected when observing the mean negative slope of the SmO_2_ reduction over the different times of measurement. This shows that the oxygen saturation of the studied muscle tissue is quickly restored after the end of exercise. Our results show that, even if the SmO_2_ reaches the initial pretreatment state, fatigue is present regarding the reduced HGS and CSHT. Therefore, the return to the initial SmO_2_ during recovery does not provide information about the full recovery time or the parameters that influence it. Therefore, monitoring SmO_2_ levels during intermittent isometric climbing-specific muscle contractions does not appear to be an appropriate means of detecting cumulative fatigue. The reduction in climbing-specific holding times did not correlate significantly with HGS between measurement times, also suggesting that HGS does not seem to be a suitable parameter for estimating athletes’ cumulative fatigue.

Fatigue means that the required hand position can no longer be maintained [23], and thus, the termination point should occur shortly thereafter. In the current study, some participants expressed this by not being able to keep their little finger on the grip of the fingerboard. Looking at the muscle oxygen metabolism, significant differences are present between pre-, termination-, and recovery- (10 sec after termination) SmO_2_ levels in connection with the onset of acute fatigue. Therefore, monitoring the muscle oxygen saturation during isometric climbing-specific holding might be useful as an indicator of acute fatigue.

Participants with relatively long CSHT seem to reach lower SmO_2_ levels at the termination time points compared to participants with lower CSHT (see Figure 6). Similarly, [14] showed, using regularly climbing participants, that the climbing-specific force production correlates negatively with the minimal attainable muscle oxygenation in the forearms in climbers in the context of HIT training. In contrast, participants with relatively long CSHT do not appear to have higher basal SmO_2_. A reason for this phenomenon could be improved muscle perfusion of the finger flexor muscles in those participants, which could lead to an improved exchange of the decisive metabolic products. Thus, baseline saturation would not be affected, but the process of metabolic exchange during exercise results in relatively longer CSHTs and lower SmO_2_ levels at the termination time points. In line with this, Ref. [22] showed that re-oxygenation was faster in finger flexor muscles of climbers during intermittent isometric tests compared to non-climbers, which also might be explained as an effect of better muscle perfusion. Matching results regarding training effects improving forearm blood flow are reported after handgrip training [24].

Participants with relatively long CSHT seem to show less variability in their SmO_2_ at the termination events. This is in accordance with the work of [15], which showed that NIRS provides a reliable measurement of oxygenation in the forearm flexors of climbers during intermittent contractions up to complete exhaustion. Therefore, SmO_2_ level seems preferable for monitoring acute fatigue in participants with relatively long CSHT. The termination SmO_2_ seems to be highly individual and possibly dependent on the training status of the participants. For monitoring acute fatigue, it seems necessary to perform an adjustment to the individual termination SmO_2_ level.

Regarding muscles involved during the measurement of the CSHT, it is stated that the *m. flexor digitorum profundus* is important in climbing, because it bends the phalanx of fingers 2–5 [22]. Some studies report that they analyzed this muscle using NIRS [14,25]. However, because the muscle lies deep and is covered by other muscles, it is questionable whether these studies could actually measure metabolic parameters of this muscle. The way in which the sensor was attached in the current study, the muscle oxygen metabolism of the muscle bellies of *m. flexor carpi radialis* and *m. palmaris longus* was especially measured. It also cannot be ruled out that other muscle groups played a greater role in CSHT. It must further be considered that the Moxy biosensor only covers a small area, whereas the finger flexors responsible for holding comprise a large portion of the forearm. Missing statistical correlations can, therefore, also be due to the isolated observation of a small muscle section. Therefore, to improve accuracy and muscle coverage, future work should also consider other sensors e.g., the OctaMon M system (Artinis Medical Systems, Elst, The Netherlands). Moreover, there are some limitations of NIRS sensors in general: [11] suggests that adipose tissues under the skin and the cutaneous blood flow when exercising could modify the results of the spectroscopy. The authors of reference [14] point out that the measurement is dependent on blood flow, oxygen consumption, and mitochondrial respiratory capacity. Finally, it should be noted that only the finger flexors were observed in climbing-specific fatigue, but in a realistic environment, other muscle groups might be fatigued before the finger flexors. Therefore, consideration should be given to performing further tests on different muscle groups (e.g., back muscles, upper arm) with a variety of different grips. Furthermore, the legs comprise a large amount of the climbers’ weight in a realistic climbing situation.

At this point, it should be stated that the training status certainly determines the CSHT significantly, but a person also achieves a long holding time simply because of low body weight [4]. Accordingly, it cannot be said in general that people with longer CSHT are better trained. The current study used the CSHT as an indicator of climbing performance as strong associations were reported in the literature [26,27]. However, it is important to keep in mind that we can only make indirect statements about the actual climbing performance with this procedure. Other methods for assessment of climbing-specific finger flexor strength, e.g., using a scale platform to measure the load that can be held by the test arm [3], are also mentioned in the literature. However, the CSHT does not necessarily represent a realistic climbing situation, as there are grip changes and short pauses between isometric contractions.

Consequently, subsequent research should also aim to analyze NIRS in the field. Furthermore, performing NIRS in combination with electromyography (EMG) might be promising in obtaining additional insights into neuromuscular factors.

## 5. Conclusions

According to the results, it is questionable whether hand dynamometers should be used for monitoring fatigue in climbing. Similarly, measurement of muscular oxygen metabolism appears to be inappropriate for monitoring cumulative fatigue in the finger flexor muscles. The results indicate that monitoring acute fatigue using athletes’ termination-SmO_2_ seems promising, especially in participants with relatively long CSHT. NIRS could therefore play an important role in monitoring acute fatigue in climbing, e.g., for improving interval training on the fingerboard by predicting the timing of muscle failure or avoiding overexertion.

## Data Availability

The data are available if there is justified research interest.
